# Protective efficacy of *Toxoplasma gondii* GRA12 or GRA7 recombinant proteins encapsulated in PLGA nanoparticles against acute *Toxoplasma gondii* infection in mice

**DOI:** 10.3389/fcimb.2023.1209755

**Published:** 2023-07-12

**Authors:** Hong-chao Sun, Pu-ming Deng, Yuan Fu, Jin-hua Deng, Rong-hui Xie, Jing Huang, Meng Qi, Tuan-yuan Shi

**Affiliations:** ^1^Institute of Animal Husbandry and Veterinary Medicine, Department of Animal Parasitology, Zhejiang Academy of Agricultural Sciences, Hangzhou, China; ^2^Institute of Animal Science and Technology, Department of Animal Diseases Diagnosis and Control of Xinjiang Production & Construction Corps, Tarim University, Alar, China; ^3^Department of Animal Epidemic Surveillance, Zhejiang Provincial Animal Disease Prevention and Control Center, Hangzhou, China

**Keywords:** GRA7, GRA12, nanoparticles, PLGA, *Toxoplasma gondii*, vaccine

## Abstract

**Background:**

Toxoplasma gondii is an apicomplexan parasite that affects the health of humans and livestock, and an effective vaccine is urgently required. Nanoparticles can modulate and improve cellular and humoral immune responses.

**Methods:**

In the current study, poly (D, L-lactic-co-glycolic acid) (PLGA) nanoparticles were used as a delivery system for the T. gondii dense granule antigens GRA12 and GRA7. BALB/c mice were injected with the vaccines and protective efficacy was evaluated.

**Results:**

Mice immunized with PLGA+GRA12 exhibited significantly higher IgG, and a noticeable predominance of IgG2a over IgG1 was also observed. There was a 1.5-fold higher level of lymphocyte proliferation in PLGA+GRA12-injected mice compared to Alum+GRA12-immunized mice. Higher levels of IFN-g and IL-10 and a lower level of IL-4 were detected, indicating that Th1 and Th2 immune responses were induced but the predominant response was Th1. There were no significant differences between Alum+GRA7-immunized and PLGA+GRA7-immunized groups. Immunization with these four vaccines resulted in significantly reduced parasite loads, but they were lowest in PLGA+GRA12-immunized mice. The survival times of mice immunized with PLGA+GRA12 were also significantly longer than those of mice in the other vaccinated groups.

**Conclusion:**

The current study indicated that T. gondii GRA12 recombinant protein encapsulated in PLGA nanoparticles is a promising vaccine against acute toxoplasmosis, but PLGA is almost useless for enhancing the immune response induced by T. gondii GRA7 recombinant protein.

## 1 Introduction

*Toxoplasma gondii* is an obligate intracellular eukaryotic parasite that can invade and replicate in almost all warm-blooded animals including mammals and birds (Dubey et al., 1970; Dubremetz, 1998). Cats (wild and domestic felids) are the only known definitive hosts for *T. gondii* and can excrete millions of environmentally resistant oocysts (Dubey, 2001). Approximately one third of people worldwide are chronically infected with *T. gongii*. Although the majority of infections in healthy adults are usually asymptomatic or have relatively mild symptoms, in immunocompromised individuals (such as AIDS patients, transplant recipients, and cancer patients) or in congenitally infected children, infection can lead to severe disease or even death (Hill and Dubey, 2002). Toxoplasmosis is one of the main causes of death in immunocompromised patients. It can be found in most parts of the world (Tenter et al., 2000), and can lead to indirect economy loss due to reproductive disorders (Boughattas et al., 2011). The life cycle of *T. gondii* is complex, there are three infectious stage of *T. gondii*: the tachyzoites, the bradyzoites (in tissue cysts) and the sporozoites (in oocysts). The sexual phase only occurs in the intestine of definitive hosts (felids) (Konradt et al., 2016). In intermediate hosts, *T. gondii* is present in two stages, rapid-replicating tachyzoites and slow-growing bradyzoites. The tachyzoites are responsible for acute infection, and the bradyzoites are responsible for chronic infection (Dubey et al., 1998; Montoya and Liesenfeld, 2004). Humans acquire infection by consuming food or water contaminated with oocysts shed from cats or raw or undercooked meat containing tissue cysts (Tenter et al., 2000).

Currently the only available toxoplasmosis vaccine is Toxovax (Intervet Inc., Angers, France), a live vaccine used for prevention in goats and sheep, which is unlikely to be used in humans (Buxton and Innes, 1995; Jongert et al., 2009). Conventional anti-*T. gondii* drugs have several disadvantages such as serious adverse side effects, limited therapeutic efficacy, emergence of drug-resistant parasites, and poor effects against *T. gondii* cysts in tissues (Zhang et al., 2013; Liu et al., 2017). Furthermore, reinfection can occur because of the complicated life cycle of the parasite (Konstantinovic et al., 2019). Hence, the development of an effective vaccine against toxoplasmosis has long been a goal. In recent years the protective effects of numerous *T. gondii* antigens have been evaluated, and the dense granule antigens (GRAs) are potential candidates for vaccines which may prevent *T. gondii* infection (Golkar et al., 2007; Hiszczyńska-Sawicka et al., 2011; Zheng et al., 2019). The presence of GRA7 in a DNA vaccine triggered strong antibody responses and higher levels of IFN-γ, indicating that GRA7 should be a main component of vaccines against *T. gondii* (Jongert et al., 2007). GRA12 is a key *T. gondii* virulence factor that resists host innate immunity triggered by IFN-γ (Fox et al., 2019). In a bioinformatics study GRA12 exhibited good antigenicity and several excellent B cell and T cell epitopes, indicating that GRA12 may be a worthwhile inclusion in a vaccine against *T. gondii* infection (Ghaffari et al., 2020).

Several synthetic delivery systems have facilitated enhanced immunogenicity of antigenic vaccine components (Jongert et al., 2009), such as Poly (D, L-lactic-co-glycolic acid) (PLGA) nanoparticles (NPs) (Allahyari and Mohit, 2016), which can be metabolized to their monomers in aqueous media and taken up by antigen presenting cells (APCs) through binding to pattern recognition molecules such as Toll like receptors (TLRs). PLGA could efficiently promote antigen targeting towards dendritic cells (DCs) and the uptake of PLGA particles by DCs can be occurred without any specific recognition because of the size of the particles were similar to the pathogens. PLGA NPs can inhibit protein degradation and prolong the release of the protein (Roozbehani et al., 2018). PLGA approved by the Food and Drug Administration (FDA) is characterized by nontoxicity, good biocompatibility and can provide sustained protein release, which is important to elicit potent immune responses (Allahyari et al., 2016; Chereddy et al., 2016). They are considered a promising *T. gondii* vaccine delivery vehicle.

In the current study vaccines containing recombinant *T. gondii* GRA7 and GRA12 proteins encapsulated in PLGA NPs were designed and administered to mice, and immunogenicity and protective efficacy were evaluated. In other mice Alum was included as an adjuvant. In fact, one candidate antigen can induce only partial protective immunity against *Toxoplasma*, and the development of a combined vaccine consisting of various stages of the parasite’s life history (tachyzoites, bradyzoites, and sporozoites) may be necessary for complete protective immunity in the future study.

## 2 Materials and methods

### 2.1 Mice

BALB/c mice aged 6–8 weeks were obtained from the Laboratory Animal Center of Zhejiang Academy of Agricultural Sciences. All protocols were approved by the Animal Care Committee of Zhejiang Academy of Agricultural Sciences in accordance with the recommendations of the National Institutes of Health Guide for the Care and Use of Laboratory Animals (Ethics protocol No. 2021ZAASLA65). All mice were kept under the conditions specified in these approved protocols.

### 2.2 Cell culture and parasite acquisition

Vero cells were cultured in Dulbecco’s Modified Eagle Medium (DMEM, Gibco, CA, USA) supplemented with 10% fetal bovine serum (FBS), 100 U/mL penicillin, and 100 μg/mL streptomycin. The cells were maintained at 37°C in a humidified incubator containing 5% CO_2_. *T. gondii* RH strain tachyzoites were maintained in Vero cells, then tachyzoites from cell cultures were collected by passing the cells through a 27-gauge needle 3–5 times. Lastly, the parasites were washed with phosphate-buffered saline (PBS) three times, then the cell fragments were filtered through a 3-μm pore membrane filter.

### 2.3 Prokaryotic expression and preparation of polyclonal antibody

Total RNA from *T. gondii* RH strain tachyzoites was obtained using TRIzol reagent in accordance with the manufacturer’s instructions (Invitrogen, USA). The cDNA was amplified *via* a reverse transcription kit (TransGen Biotech, China). The CDS sequence of GRA12 without the hydrophobic signal peptide was amplified *via* the polymerase chain reaction (PCR) with primers, 5’-GCGAATTCATGCGACATGTTGGCGGTTTCTCGG-3’ (forward), 5’-CCAAGCTTTCAGTTGTGTTTGCTGCCTGCAGAG-3’ (reverse). The primers contain *EcoRI* and *HindIII* restriction sites (underlined). PCR product was recovered using a DNA purification kit and constructed into the pEASY-T1 vector (TransGen Biotech, China). The clones were sequenced (Sangon Biotech, China), then constructed into the expression vector pET32-a (Takara, Japan). The recombinant plasmid was transferred into *Escherichia coli* BL21 cells (Takara, Japan), then bacterial cultures were induced by 1 mM Isopropyl-beta-D-thiogalactopyranoside (IPTG) and the recombinant protein was identified by sodium dodecyl sulfate-polyacrylamide gel electrophoresis (SDS-PAGE) with a 12% polyacrylamide gel. Western blotting analyses were performed to verify the structure of the recombinant protein. GRA12-pET32a-his fusion protein was then purified using Ni-NTA purification agarose (GE, USA).

he protein was injected intraperitoneally into mice aged 6–8 weeks to generate polyclonal antibody. Briefly, the protein was fully emulsified with Freund’s complete adjuvant (Thermo Fisher Scientific, USA) at a ratio of 1:1 and the injection dose was 100 μg/mouse for the first immunization. For the second and third immunizations the protein was emulsified with Freund’s incomplete adjuvant (Thermo Fisher Scientific, USA) and the injection dose was 50 μg/mouse. After the final immunization antibody titers were analyzed *via* western blotting and enzyme-linked immunosorbent assays (ELISAs). Recombinant GRA7 protein was obtained as described above, and the primers used were 5’-GGGGTACCATGGCCACCGCGTCAGATGACG-3’ (forward) and primer, 5’-CGGATAT *C* CTGGCGGGCATCCTCCCCATC-3’ (reverse), with restriction enzyme sites *KpnI* and *EcoRV* (underlined).

### 2.4 Preparation of different NPs

With minor modifications, PLGA NPs were prepared using the water/oil/water (w/o/w) solvent evaporation technique (Jeffery et al., 1993). Briefly, 10 mg of PLGA was dissolved in 1 mL of dichloromethane (DCM) to obtain a uniform PLGA solution (w/o), and this solution was sonicated at 30 W for 10 min (run for 3 s and pause for 3 s). Then, 10 mL of 2% (w/v) polyvinyl alcohol (PVA) was dissolved dropwise in PLGA solution, and the PLGA/PVA (w/o/w) solution was emulsified for 10 min as above. Subsequently, 0.5% PVA was added to a final volume of 20 mL with continuous stirring at 400 rpm on a magnetic stirrer at room temperature for 4 h to completely remove DCM. After centrifugation at 14,000 *g* for 30 min at 4°C the NPs were collected and dissolved in distilled water. The PLGA NPs with recombinant proteins (*T. gondii* GRA7 and *T. gondii* GRA12) and non-proteins were prepared together. The recombinant GRA7 and GRA12 proteins were used at a concentration of 1 mg/mL, and the supernatant was also collected to calculate the total amount of protein. All prepared NPs were stored at -80°C until use.

### 2.5 NPs characterization and encapsulation efficiency

A dynamic light-scattering instrument (Anton Paar Litesizer 500) and transmission electron microscopy (TEM) were used to determine the average size and distribution of NPs. A total of 5 mg of recombinant *T. gondii* GRA7 or *T. gondii* GRA12 protein was encapsulated in NPs as described above. Concentrations of unbound protein were assessed using a BCA protein assay kit (Sangon Biotech, China). Encapsulation efficiency (EE), (LC), and cumulative release (CR) were determined using the following equations:


EE%=(Total amount of protein–free protein)/Total amount of protein×100%

LC%=Total amount of encapsulated protein/Total amount of NPs×100%

CR%=(Total volume of supernatant×protein concentration)/Total amount of loaded protein×100%

### 2.6 Immunization and challenge

Seven groups of female BALB/c mice (13 mice/group) were intraperitoneally immunized with PLGA-NPs-TgGRA7 (100 μg/mouse), PLGA-NPs-TgGRA12 (100 μg/mouse), TgGRA7 emulsified in Aluminum hydroxide (100 μg/mouse) (Sigma Aldrich, Germany), TgGRA12 emulsified in Aluminum hydroxide (100 μg/mouse), Aluminum (100 μL/mouse), PLGA-NPs (100 μg/mouse), or PBS (100 μL/mouse), and an eighth group was left unimmunized. For second and third immunizations the amount of protein administered was 50 μg/mouse, and the interval between each immunization was 14 d. Five weeks after the last immunization 10 mice from each group were challenged with 1 × 10^3^ *T. gondii* RH strain tachyzoites. The status of infected mice was monitored each day, and the survival time of each mouse was recorded daily.

### 2.7 Preparation of *T. gondii* lysate antigen

*T. gondii* lysate antigen (TLA) was prepared as described previously with minor modifications (Holec-Gasior et al., 2010). Briefly, tachyzoites were collected and washed with sterile PBS three times, then centrifuged at 1000 rpm for 10 min, and the supernatant was discarded. The precipitation was then dissolved in 3 mL PBS and sonicated for 10 min at 70 W/s on ice, then the tachyzoites were repeatedly freeze-thawed at –80°C and 37°C. Lastly, the concentration of TLA was measured using a BCA protein assay kit, and the TLA was stored at –80°C until use.

### 2.8 Spleen cell proliferation and cytokine assays

Three mice from each group were euthanized 5 weeks after the last immunization, and their spleens were collected. A splenocyte suspension was passed through a mesh sieve and treated with erythrocyte lysate buffer to remove red blood cells (Solarbio, Beijing, China). The splenocytes were then resuspended in DMEM medium supplemented with 10% FBS, 100 U/mL penicillin, and 100 μg/mL streptomycin. Next, the spleen cells were cultured in a 96-well plate at a concentration of 1 × 10^5^ cells/well and incubated with 20 μg/mL TLA (Roozbehani et al., 2018), or 7.5 μg/mL concanavalin A (ConA, Sigma, USA) as a positive control, or DMEM medium as a negative control. After culturing at 37°C with 5% CO_2_ for 72 h, splenocyte proliferation was analyzed using cell counting kit-8 (Biosharp, Anhui, China). Average optical density (OD) at 450 nm was measured using a microplate reader. All samples were run in triplicate.

The supernatants of splenocytes stimulated with TLA were collected at 24, 48 and 96 h, and cytokines were detected using ELISA kits in accordance with the manufacturer’s instructions (MultiSciences, Hangzhou, China). The experiments were done in triplicate. Cytokine concentrations were calculated by referring to standard curves, and the data were expressed as means ± SDs for each group.

### 2.9 Measurement of antibody responses

Blood samples were collected from the tail vein for each group (three mice/group) 5 weeks after the last immunization, and antibody levels (IgG, IgG1, and IgG2a) were assessed *via* ELISAs in accordance with the manufacturer’s instructions (MultiSciences, Hangzhou, China). Briefly, 96-well plates were coated with TLA (20 μg/mL) overnight at 4°C, then washed three times with PBS containing 0.05% Tween 20 (PBST). The plates were then blocked with 1% bovine serum albumin (BSA) for 2 h at 37°C. After washing with PBST, the sera (diluted 1:100 using PBS-1%BSA) were incubated in the wells for 2 h at 37°C. After washing, horseradish peroxidase-conjugated goat anti-mouse IgG (diluted 1:1000 in PBS-1%BSA), anti-mouse IgG1 (diluted 1:1000 in PBS-1%BSA), or anti-mouse IgG2a (diluted 1:1000 in PBS-1%BSA) were added to the wells and incubated for 1 h at 37°C. After washing with PBST three times, the immune complexes were developed by incubation with tetramethylbenzidine (TMB) substrate solution for 15 min at 37°C, and the reaction was terminated by using 2 M H_2_SO_4_. Absorbance was determined at 450 nm. All tests were performed in triplicate.

### 2.10 Determination of parasite loads

To evaluate parasite loads, heart, liver, spleen, and lung tissues were collected from three mice in each group that exhibited clinical symptoms but did not die. Genomic DNA was extracted using a tissue DNA extraction kit (Coolaber, Beijing, China) in accordance with the manufacturer’s instructions. Parasite loads were determined *via* SYBR-green quantitative real-time PCR (Q-PCR) using the repeated element (RE) primers 5’-AGGGACAGAAGTCGAAGGGG-3’ (forward) and 5’-GCAGCCAAGCCGGAAACATC-3’ (reverse). Q-PCR was performed with an Applied Biosystems Inc. 7500 fluorescence quantitative PCR instrument. The protocol used was initial denaturation at 95°C for 10 min, followed by 40 cycles of denaturation at 95°C for 15 s, annealing at 60°C for 30 s, and amplification at 72°C for 30 s. Melting curve analysis was performed to estimate the specific amplification of the primers. The standard curve was obtained *via* amplification of the target sequence from the genomic DNA of *T. gondii* tachyzoites, and parasite loads were calculated based on the standard curve (Y = -2.62X + 30.206; R^2 = ^0.996). The data represent three independent experiments.

### 2.11 Statistical analysis

Statistical analyses were performed using GraphPad Prism version 5 (GraphPad Prism, San Diego, CA, USA). One-way analysis of variance was used to analyze differences in antibody and cytokine levels, the Kaplan–Meier approach was used to assess survival. The *t*-test was used to compare means between two groups. *p*< 0.05 was deemed to indicate statistical significance.

## 3 Results

### 3.1 Recombinant protein expression

**Figure 1:**
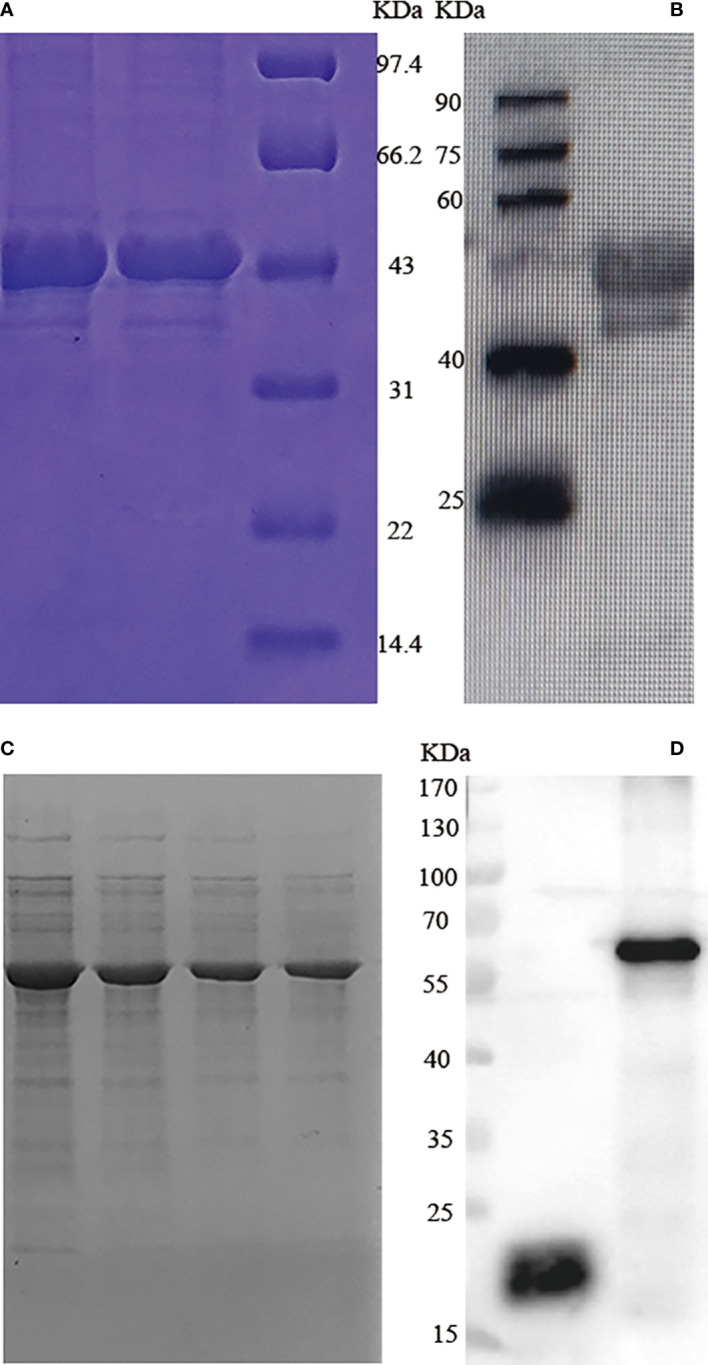
SDS-PAGE and western blotting analysis of *T. gondii* GRA7 and GRA12 recombinant proteins. **(A)** SDS-PAGE analysis of recombinant *T. gondii* GRA7 protein purified *via* Ni-NTA purification agarose. **(B)** Western blotting analysis of recombinant *T. gondii* GRA7-pET-32a protein. **(C)** SDS-PAGE analysis of recombinant *T. gondii* GRA12 protein purified *via* Ni-NTA purification agarose. **(D)** Western blotting analysis of recombinant *T. gondii* GRA12 pET-32a protein. The left band represents pET-32a, and the right band represents the recombinant *T. gondii* GRA12- pET-32a.

GRA7 and GRA12 target products were observed as single bands of approximately 45 kDa (GRA7) and 61 kDa (GRA12) on SDS-PAGE (**Figures 1A, C**). In western blotting analysis recombinant TgGRA7 and TgGRA12 proteins were recognized by mouse anti-*T. gondii* serum polyclonal antibodies (**Figures 1B, D**).

### 3.2 Physical characterization of NPs and release characteristics

**Figure 2:**
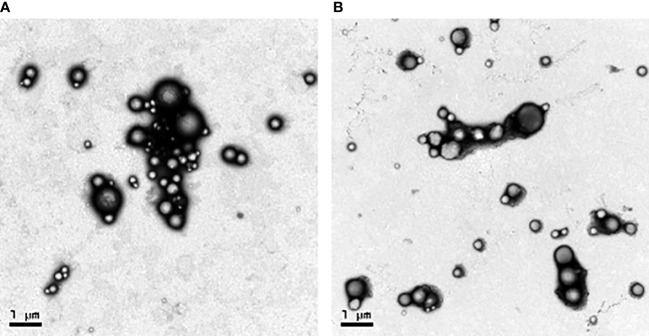
Sizes and distributions of recombinant *T. gondii* GRA7 and GRA12 proteins encapsulated in PLGA NPs as observed *via* TEM. **(A)** PLGA+GRA7 NPs (10,000x, scale bar = 1 μm.) **(B)** PLGA+GRA12 NPs (10,000x, scale bar = 1 μm).

**Figure 3:**
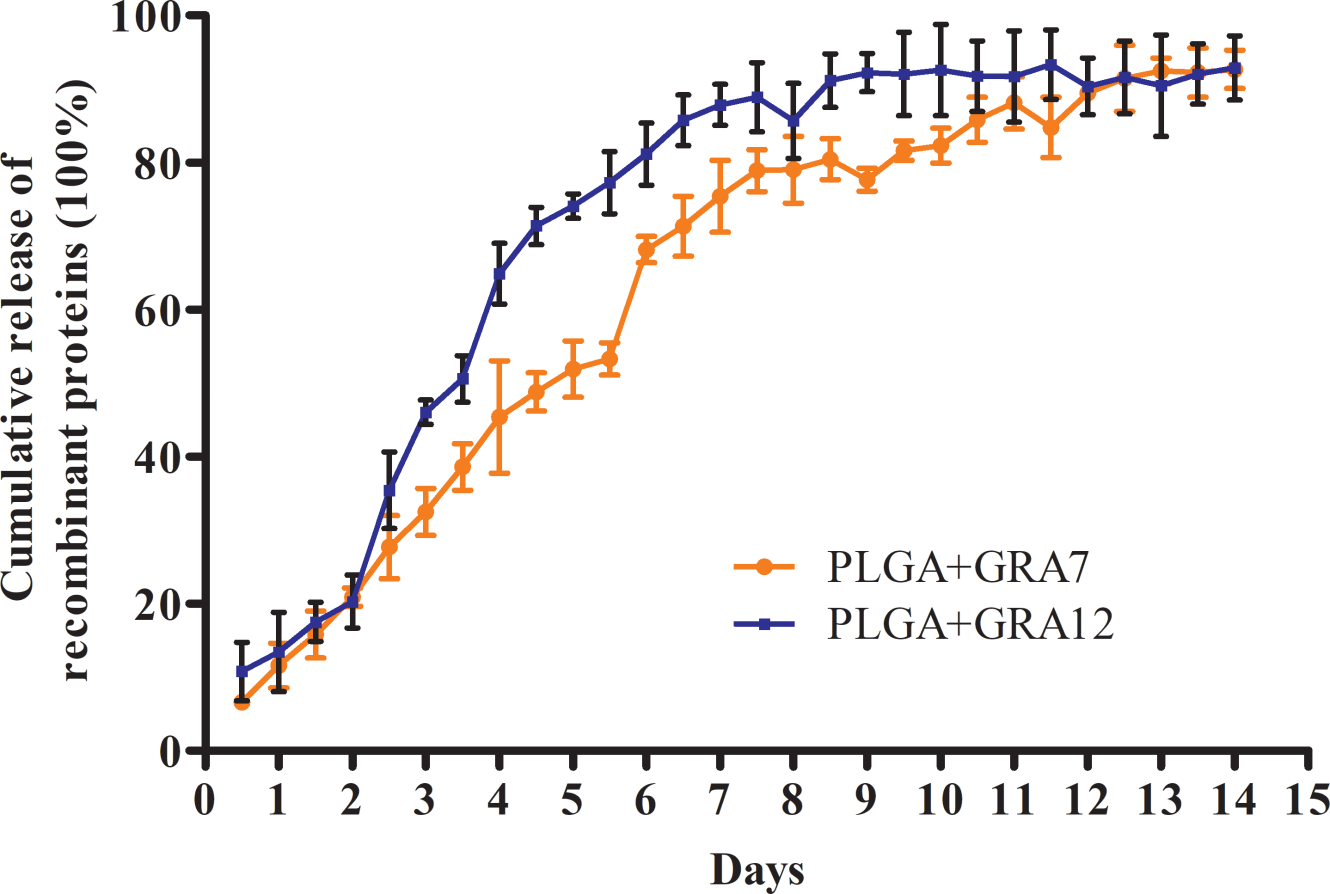
Release characteristics of *T. gondii* GRA7 and GRA12 proteins using PLGA NPs as a delivery system. Data represent mean ± SD, *n* = 3 experiments.

Transmission electron microscopy (TEM) images revealed that the NPs were spherical with a smooth surface (**Figures 2A, B**). The mean NP diameter of PLGA+GRA12 was 106.0 ± 22.1 nm, and the mean diameter of PLGA+GRA7 was 130.8 ± 27.1 nm. The initial release of GRA7 protein from PLGA+GRA7 NPs was 6.6% ± 0.5%, and the initial release of GRA12 protein from PLGA+GRA12 NPs was 10.8% ± 3.4%. Antigen release was highest during the first week (GRA7 78.9% ± 2.3%, GRA12 88.9% ± 3.8%), then it slowly increased. In the following days the cumulative release of the recombinant proteins tended to be low, and ultimately 92.7% ± 2.1% of GRA7 and 92.9% ± 3.6% of GRA12 were released (**Figure 3**).

### 3.3 Antibody secretion in mice

**Figure 4:**
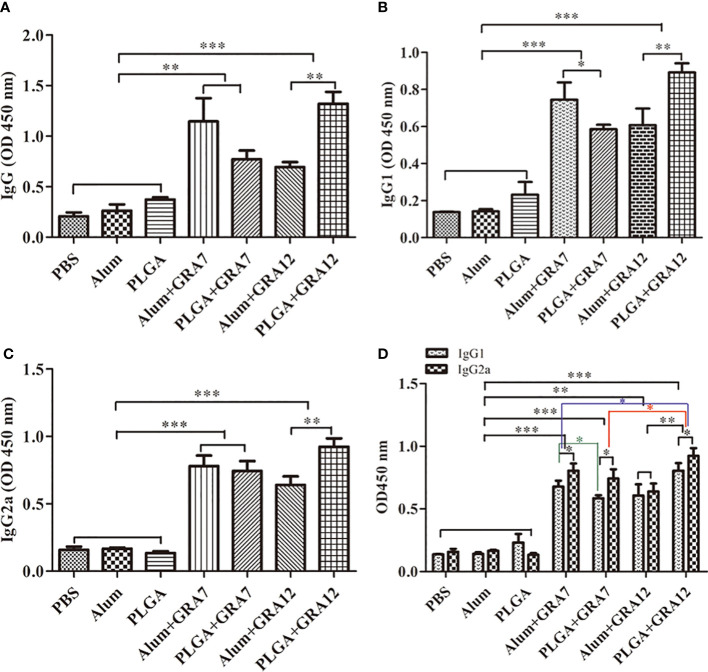
Specific antibody responses in mice immunized with Alum+GRA7, PLGA+GRA7, Alum+GRA12, PLGA+GRA12, PBS, Alum, and PLGA. **(A)** IgG secretion levels. **(B)** IgG1 secretion levels. **(C)** IgG2a secretion levels. **(D)** Levels of IgG1 and IgG2a. Results are represented as 450 nm optical density means ± SD. ^*^p< 0.05, ^**^p< 0.01, ^***^p< 0.001.

Compared with the PBS, Alum alone, and PLGA alone control groups, levels of IgG were significantly higher in the in the Alum+GRA7 and PLGA+GRA7 group (*p*< 0.01) and the Alum+GRA12 and PLGA+GRA12 groups (*p*< 0.001). Levels of IgG were significantly higher in the PLGA+GRA12 group than in the Alum+GRA12 group (*p*< 0.01), whereas there was no significant difference between the Alum+GRA7 group and the PLGA+GRA7 group (*p* > 0.05) (**Figure 4A**). Alum+GRA12, PLGA+GRA12, Alum+GRA7, and PLGA+GRA7 induced significantly higher IgG1 and IgG2a titers than those of the PBS, Alum alone, and PLGA alone control groups (*p*< 0.001). All vaccine-immunized groups generated higher levels of IgG1 and IgG2a than the control groups (**Figure 4B**). IgG2a levels were significantly higher than IgG1 levels in the PLGA+GRA12, Alum+GRA7, and PLGA+GRA7 groups (*p*< 0.05) (**Figure 4C**). IgG2a/IgG1 ratios were significantly higher in all vaccine-immunized groups than in the three control groups. IgG2a levels were higher in the PLGA+GRA12 group than in all other vaccine-immunized groups (*p*< 0.05), and there was a significant predominance of IgG2a over IgG1 in the PLGA+GRA12 group (*p*< 0.05) (**Figure 4D**). The detailed data were presented in **Table 1**. Collectively, the results indicated that a predominant Th1 immune response was elicited, especially in the PLGA+GRA12 immunization group.

**Table 1:** Levels of IgG, IgG1 and IgG2a antibody in sera from BALB/c mice.

Group	OD450 nm		
	IgG	IgG1	IgG2a
PBS	0.21±0.03	0.14±0.00	0.16±0.02
Alum	0.26±0.04	0.14±0.00	0.17±0.01
PLGA	0.37±0.01	0.23±0.05	0.13±0.01
Alum+GRA7	1.15±0.16 **a**	0.74±0.07 **d**	0.78±0.06 **h**
PLGA+GRA7	0.77±0.06 **a**	0.59±0.02 **d, f**	0.74±0.05 **h**
Alum+GRA12	0.69±0.04 **b**	0.61±0.06 **e**	0.64±0.04 **i**
PLGA+GRA12	1.32±0.08 **b, c**	0.89±0.03 **e, g**	0.92±0.04 **i, j**

**a**, compared with PBS, Alum or PLGA, p < 0.01.

**b**, **d**, **e**, **h**, **i**, compared with PBS, Alum or PLGA, p < 0.001.

**c**, **g**, **j**, compared with Alum+GRA12, p < 0.01.

**f**, compared with Alum+GRA7, p < 0.05.

### 3.4 Lymphocyte proliferation

**Figure 5:**
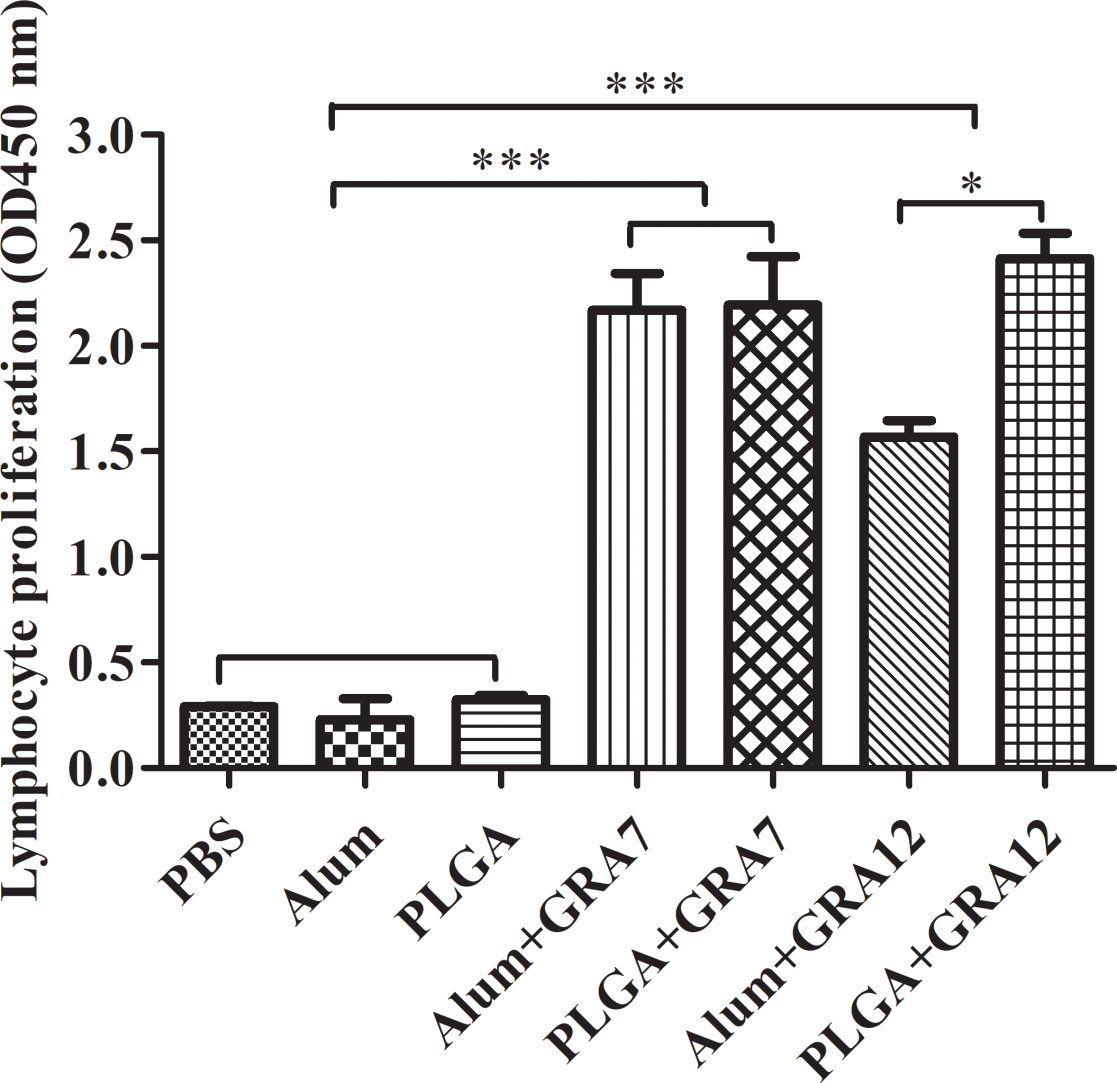
Lymphocyte proliferation was evaluated 5 weeks after the last immunization. Five weeks after the last immunization spleens were collected and lymphocyte proliferation responses were assessed. Results represent 450-nm optical density means ± SD. ^*^p< 0.05, ^***^p< 0.001.

Splenocytes from vaccine-immunized groups exhibited significantly greater proliferation than splenocytes from the control groups (*p*< 0.001). Proliferation in the PLGA+GRA12 group was almost 1.54-fold higher than that in the Alum+GRA12-immunized group (*p*< 0.05). Proliferation in the Alum+GRA7 and PLGA+GRA7 groups was significantly greater than that in the control groups (*p*< 0.001), but the difference between these two experimental groups was not significant (*p* > 0.05) (**Figure 5**). The PLGA+GRA12 group exhibited the highest lymphocyte proliferation. Data were shown in **Table 2**.

**Table 2:** Analysis of lymphocyte proliferation from BALB/c mice.

Group	OD450 nm
PBS	0.29±0.01
Alum	0.23±0.12
PLGA	0.32±0.02
Alum+GRA7	2.17±0.21 **a**
PLGA+GRA7	2.19±0.28 **a**
Alum+GRA12	1.57±0.10 **b**
PLGA+GRA12	2.41±0.14 **b, c**

**a**, **b**, compared with PBS, Alum or PLGA, p < 0.001.

**c**, compared with Alum+GRA12, p < 0.05.

### 3.5 Splenocyte cytokine production

**Figure 6:**
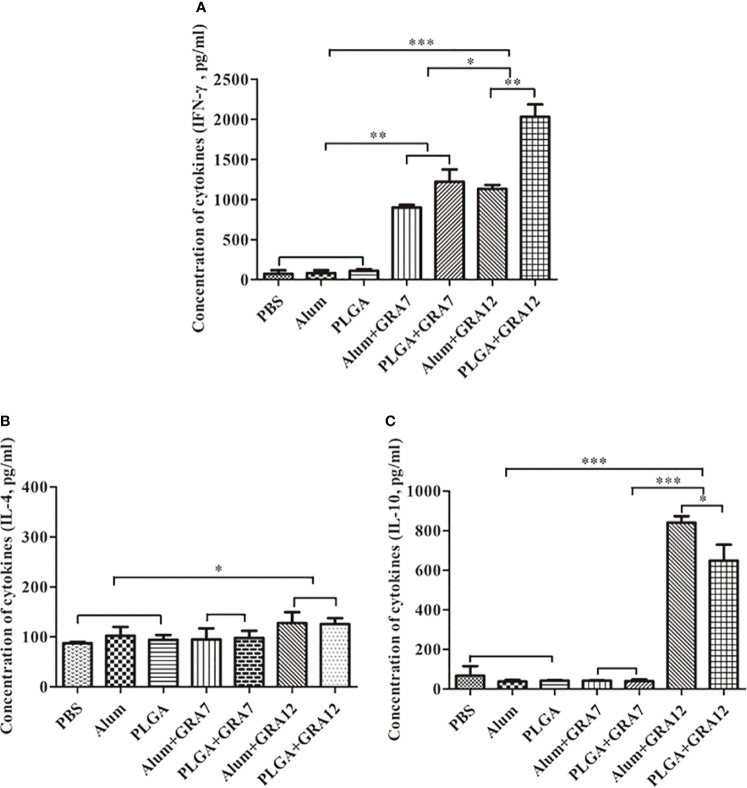
Splenocyte cytokine production after *T. gondii* lysate TLA stimulation. **(A)** IFN-γ. **(B)** IL-4. **(C)** IL-10. Three mice from each group were analyzed. Data represent means ± SD of three independent experiments. ^*^p< 0.05, ^**^p< 0.01, ^***^p< 0.001.

Compared to the control groups, splenocyte culture IFN-γ levels were significantly higher in the Alum+GRA12 and PLGA+GRA12 groups (*p*< 0.001), and in the Alum+GRA7 and PLGA+GRA7 groups (*p*< 0.01). Splenocyte culture levels of IFN-γ in the Alum+GRA12 and PLGA+GRA12 groups were higher than those in the Alum+GRA7 and PLGA+GRA7 groups (**Figure 6A**). The mean IFN-γ level in the PLGA+GRA12 group was 2030.50 pg/mL, which was significantly higher than that in the Alum+GRA12 group (1133.14 pg/mL) (*p*< 0.01). The mean IFN-γ level in the PLGA+GRA7 group was 1219.44 pg/mL and that in the Alum+GRA7 was 898.31 pg/mL, and the difference was not significant (*p* > 0.05). Splenocyte culture IL-4 was significantly higher in the Alum+GRA12 and PLGA+GRA12 groups compared to the control groups (*p*< 0.05) (**Figure 6B**). The Alum+GRA12 and PLGA+GRA12 group splenocytes generated higher levels of IL-10 than splenocytes from the other vaccine-immunized groups and the control groups (*p*< 0.001). Levels of splenocyte culture IL-10 in the Alum+GRA12-immunized group were significantly greater than those in the PLGA+GRA12 group (*p*< 0.05) (**Figure 6C**). The detailed data were shown in **Table 3**.

**Table 3:** Cytokine production in the spleen from BALB/c mice.

Group	Cytokine production (pg/mL)		
	IFN-γ	IL-4	IL-10
PBS	69.11±9.81	87.33±1.88	66.90±3.43
Alum	81.19±16.75	102.33±12.41	37.50±5.87
PLGA	108.44±23.78	94.04±6.92	42.22±1.45
Alum+GRA7	898.31±40.87 **a**	94.81±15.47	42.14±1.03 **f**
PLGA+GRA7	1219.44±190.11 **a**	97.85±10.22	39.69±5.94 **f**
Alum+GRA12	1133.14±56.84 **b, c**	127.52±15.63 **e**	840.44±23.29 **g**
PLGA+GRA12	2030.50±187.32 **b, c, d**	125.48±8.46 **e**	648.53±57.02 **g, h**

**a**, compared with PBS, Alum or PLGA, p < 0.01.

**b**, **f**, **g**, compared with PBS, Alum or PLGA, p < 0.001.

**c**, compared with Alum+GRA7 and PLGA+GRA7, p < 0.05.

**d**, compared with Alum+GRA12, p < 0.01.

**e**, compared with PBS, Alum or PLGA, p < 0.05.

**h**, compared with Alum+GRA12, p < 0.05.

### 3.6 Parasite loads in mice

**Figure 7:**
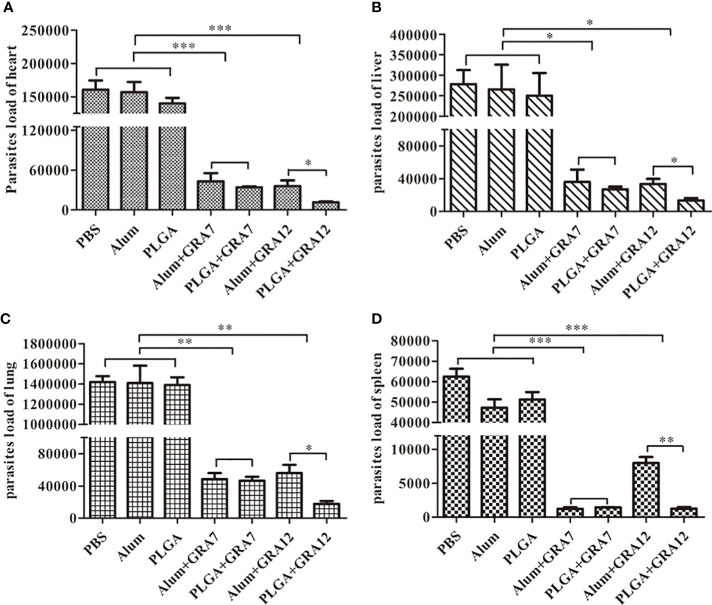
Parasite loads were analyzed using SYBR-green quantification real-time PCR. Heart, liver, lung, and spleen tissues were collected when symptoms of *T. gondii* infection became evident, and parasite loads were evaluated. **(A)** Parasite loads in the heart. **(B)** Parasite loads in the liver. **(C)** Parasite loads in the lung. **(D)** Parasites loads in the spleen. Data are presented as means ± SD of three independent experiments. *p<0.05, **p< 0.01, ***p<0.001.

Compared to mice in the PBS, Alum alone, and PLGA alone control groups, mice in the Alum+GRA7, PLGA+GRA7, Alum+GRA12, and PLGA+GRA12 groups exhibited significantly less parasites in the heart (*p*< 0.001), spleen (*p*< 0.001), lungs (*p*< 0.01), and liver (*p*< 0.01) (**Figures 7A-D**). Compared with the Alum+GRA12-vaccinated group, in the PLGA+GRA12 group the mean parasite loads were 3.08-fold lower in the heart, 2.49-fold lower in the liver, 3.16-fold lower in the lung (all *p*< 0.05), and 6.30-fold lower parasite loads in the spleen (*p*< 0.01). There were significant no differences between the PLGA+GRA7 group and the Alum+GRA7 group (*p* > 0.05). The detailed data were shown in **Table 4**.

**Table 4:** Parasite loads of heart, liver, lung and spleen from BALB/c mice.

Group	parasite loads of tissues (×10^5^)^a^			
	heart	liver	lung	spleen
PBS	1.61±0.17	2.78±0.42	14.19±0.72	0.62±0.05
Alum	1.57±0.19	2.66±0.74	14.09±2.13	0.47±0.05
PLGA	1.40±0.10	2.50±0.68	13.90±0.95	0.51±0.04
Alum+GRA7	0.43±0.15 **b**	0.36±0.09 **e**	0.48±0.09 **h**	0.01±0.00 **k**
PLGA+GRA7	0.34±0.01 **b**	0.27±0.04 **e**	0.47±0.06 **h**	0.01±0.00 **k**
Alum+GRA12	0.36±0.10 **c**	0.33±0.08 **f**	0.56±0.13 **i**	0.08±0.01 **l**
PLGA+GRA12	0.12±0.02 **c, d**	0.13±0.03 **f, g**	0.18±0.04 **i, j**	0.01±0.00 **l, m**

**a**, the average value and standard deviation represented ×10^5^.

**b**, **c**, **k**, **l**, compared with PBS, Alum or PLGA, p < 0.001.

**d**, **g**, **j**, compared with Alum+GRA12, p < 0.05.

**e**, **f** compared with PBS, Alum or PLGA, p < 0.05.

**h**, **i**, compared with PBS, Alum or PLGA, p < 0.01.

**m**, compared with Alum+GRA12, p < 0.01.

### 3.7 Vaccine-induced protection

**Figure 8:**
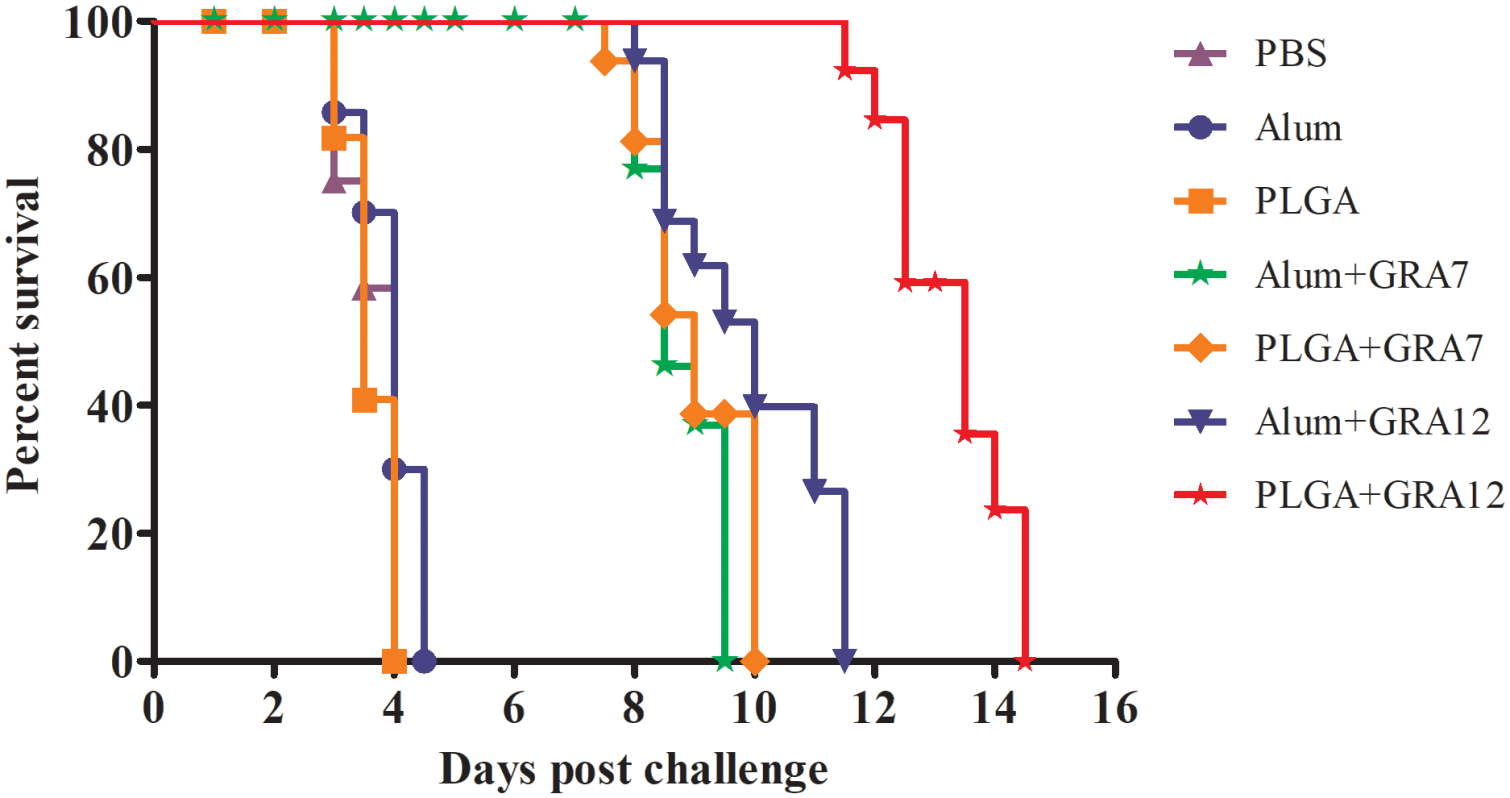
Survival curves of immunized BALB/c mice after *T. gondii* challenge. Mice were challenged with 1 × 10^3^ *T. gondii* RH strain tachyzoites 5 weeks after the final immunization (*n* = 10 per group).

Mice in the PBS, Alum alone, and PLGA alone control groups began dying on day 3, and they were all dead within 4.5 days (**Figure 8**). Compared to the control groups, survival times were significantly longer in the Alum+GRA7, PLGA+GRA7, and Alum+GRA12 groups (*p*< 0.05), and they were dramatically longer in the PLGA+GRA12 group (14.5 days; *p*< 0.01). Survival times were significantly longer in the PLGA+GRA12 group than in the Alum+GRA12 group (mean 9.5 days) (*p*< 0.05). Mean survival time was longer in the PLGA+GRA7 group than in the Alum+GRA7 group, but the difference was not statistically significant.

## 4 Discussion

Vaccines are considered the most effective tools against *T. gondii* infection, and recombinant immunogenic proteins have predominated in the quest to prevent toxoplasmosis. Nanocarrier delivery systems can facilitate the development of novel vaccines (Naeem et al., 2018), and overcome the disadvantage of short-lasting immune responses associated with some tradition vaccines. PLGA is currently the most widely used biodegradable material used to construct carriers of drugs (Shi et al., 2020), bacterial or viral DNA (Koerner et al., 2019; Lu et al., 2020), and proteins (Zhai et al., 2020). PLGA NPs can enhance immune response by continually releasing antigens, reduce protein degradation, and maintaining protein/drug levels at effective concentrations over a long period (Demento et al., 2011; Allahyari and Mohit, 2016). The sustained release of antigens may promote their uptake by APCs, and in the current study significantly long-lasting immune responses against *T. gondii* infection were induced (Lim et al., 2009).

In the present study IgG levels the Alum+GR7, PLGA+GRA7, Alum+GRA12, and PLGA+GRA12 groups were higher than those in the PBS, Alum alone, and PLGA alone control groups. Specific IgG antibody can reduce the capacity of *T. gondii* to adhere to and invade host cells, effectively blocking the connection between the parasite and the host (Kang et al., 2000). Sera of mice immunized with Alum+GR7, PLGA+GRA7, Alum+GRA12, and PLGA+GRA12 exhibited high concentrations of IgG2a, and a high IgG2a:IgG1 ratio was evident in the PLGA+GRA12 group. IgG2a is generally associated with Th1 immune responses, and IgG2a is reportedly an important component of immunity against *T. gondii* infection (Guo et al., 2018). Although mice in the GA12+PLGA group generated both higher IgG1 and IgG2a, the high IgG2a:IgG1 ratio indicated that the dominant immune response was Th1-type. In a previous study both IgG1 and IgG2a were induced in mice vaccinated with CS-SAG1-NLT, and IgG2a production predominated (Khorshidvand et al., 2022). In another study mice vaccinated with pVAX1-SIR2-CS (chitosan) exhibited a mixed Th1/Th2 immune response, suggesting that certain types of nanospheres could induce a mixed immune response (Yu et al., 2021b).

In the current study all four experimental vaccine preparations induced strong lymphoproliferative responses, but that in the PLGA+GRA12 group was significantly stronger than that in the Alum+GRA12-immunization group, and there was no significant difference between the Alum+GRA7 group and the PLGA+GRA7 group. In a previous study higher levels of splenocyte proliferation were observed in TgSORO-pVAX1/PLGA-vaccinated mice (Yu et al., 2021a). Ahmadpour et al. (2017) reported that a *T. gondii* vaccine coated with a nano-adjuvant (“GRA14+CaPN”) enhanced lymphocyte proliferation compared to the same vaccine without CaPN (Ahmadpour et al., 2017). In another previous study strong long-lasting lymphocyte proliferation was evident in mice vaccinated with PLG-rSAG1/2 microparticles (Chuang et al., 2017).

IFN-γ is an integral component of Th1-type immune responses, which play a vital role during the acute phase of *T. gondii* infection (Suzuki et al., 1988). It can activate a series of genes such as inducible nitric oxide synthase (iNOs) and inducibly expressed GTPase (IGTPs) which can inhibit the growth of *T. gondii* (Der et al., 1998; Taylor et al., 2000). Th2-type cytokines such as IL-4 can inhibit severe immunopathology during *T. gondii* infection, and IL-4 can also counterbalance the immune response by downregulating IFN-γ secretion (Harris et al., 2005; Hunter and Sibley, 2012), which is consistent with the high IFN-γ and low IL-4 observed in in the present study. IL-10 has a prominent role in the prevention of immune hyperactivity (Wilson et al., 2005), and it inhibits the activity of Th1 cells, thus maintaining immune balance by reducing inflammatory reactions during toxoplasmosis (Dupont et al., 2012). In a previous study IL-10 secretion levels were increased in animals injected with a pVAX1-SIR2/PLGA vaccine (Yu et al., 2021b), and IL-10 was observed at high levels in rTgPLP2-immunized mice (Tian et al., 2021). In that same study mice exhibited a lethal T cell-mediated response due to excessive levels of IFN-γ in the absence of IL-10 (Suzuki et al., 2000; Wille et al., 2001). In the present study IL-10 secretion from splenocytes of mice in the Alum+GRA12 and PLGA+GRA12 groups was evident, which may indicate excessive humoral immunity. Although protection against *T. gondii* infection mainly depends on cellular immunity, humoral immunity also plays an indispensable role (Lu et al., 2018).

*T. gondii* parasite loads in tissues have been detected *via* Q-PCR in many studies (Dadimoghaddam et al., 2014; Montazeri et al., 2016), and the method is evidently highly specific and sensitive. Fatollahzadeh et al. (2021) found that parasite loads were significantly reduced in the spleens and brains of pcGRA14+pcROP13-immunized mice (Fatollahzadeh et al., 2021). In another study parasitic burden was reduced in all tissues investigated in pcGRA14-CaPN-immunized mice (Yu et al., 2021a). In the current study parasite loads were significantly reduced in the experimental vaccination groups compared to the control groups, and parasitic burden in the PLGA+GRA12 group was significantly reduced compared to the Alum+GRA12 group. There was no significant difference between the Alum+GRA7 and PLGA+GRA7 groups. The results of the present study indicate that PLGA NPs can enhance the immune protective effects of recombinant *T. gondii* GRA12, but are ineffective with respect to recombinant *T. gondii* GRA7. Numerous studies have confirmed that NP adjuvants enhance immune responses and increase the immunogenicity of vaccines (Roozbehani et al., 2018; Hasan et al., 2021), and PLGA is biocompatible and may generate robust immunity against toxoplasmosis (Zhang et al., 2016).

Monitoring the survival rate of mice infected with *T. gondii* is currently considered the best way to evaluate the efficiency of candidate *T. gondii* vaccines (Zheng et al., 2017). In the current study, all four experimental vaccines exhibited extended survival time compared to mice in the control groups. Survival rates in the Alum+GRA7, PLGA+GRA7, and Alum+GRA12 groups were lower than that in the PLGA+GRA12 group, and significantly prolonged survival was evident in the PLGA+GRA12 group. In a previous study, mice vaccinated with rTgH2A1/PLGA exhibited a significantly increased survival time (Yu et al., 2020). In future experiments, it would be informative to evaluate the immune protection induced by PLGA+GRA12 in low-virulence *T. gondii* strains.

In conclusion, in the current study vaccination with *T. gondii* GRA12 encapsulated in PLGA NPs triggered strong humoral and cellular immune responses, reduced parasite loads, and prolonged survival time after *T. gondii* infection in BALB/c mice.

## Data availability statement

The raw data supporting the conclusions of this article will be made available by the authors, without undue reservation.

## Ethics statement

All experiments performed on animals were approved by the Animal welfare and Ethics Committee of Zhejiang Academy of Agricultural Sciences.

## Author contributions

H-cS and T-yS designed the study and revised the manuscript. H-cS drafted the manuscript. H-cS and T-yS supported the study. P-mD, YF, and J-hD performed the experiments for preparation of recombinant proteins, polyclonal antibody and vaccines. R-hX and JH performed the animal experiment and immune protection analysis. MQ analyzed the data. All authors have read and agreed with the submitted version. All authors contributed to the article.
